# The Prevalence of Diabetes Mellitus in COPD Patients with Severe and Very Severe Stage of the Disease

**DOI:** 10.3889/oamjms.2016.060

**Published:** 2016-05-22

**Authors:** Jagoda Stojkovikj, Beti Zafirova-Ivanovska, Biserka Kaeva, Sasha Anastasova, Irena Angelovska, Smiljko Jovanovski, Dragana Stojkovikj

**Affiliations:** 1 *University Clinic of Pulmollogy and Allergology, Faculty of Medicine, Ss Cyril and Methodius University of Skopje, Skopje, Republic of Macedonia*; 2 *Institute for Epidemiology and Biostatistics and Medical Informatics, Faculty of Medicine, Ss Cyril and Methodius University of Skopje, Skopje, Republic of Macedonia*; 3 *University Clinic of Cardiology, Faculty of Medicine, Ss Cyril and Methodius University of Skopje, Skopje, Republic of Macedonia*; 4 *School of Doctoral Studies at Faculty of Medicine, Ss Cyril and Methodius University of Skopje, Skopje, Republic of Macedonia*

**Keywords:** severe COPD, very severe COPD, comorbidity, prevalence of diabetes mellitus type 2

## Abstract

**AIM:**

The aim of the study was to investigate the prevalence of diabetes mellitus in privies diagnosed chronic obstructive pulmonary disease (COPD) patients with severe and very severe disease, which ware stable.

**METHODS:**

We investigated 100 subjects, all of them smokers, with smoking status >10 years. They were stratified in two groups. It was clinical, randomized, cross sectional study. Besides demographic parameters, functional parameters, BMI, cholesterol, LDL and HDL, and the level of blood sugar was measured.

**RESULTS:**

The prevalence of diabetes mellitus in our survey in total number of COPD patients with severe and very severe stage was 21%. In the very severe group were recorded significantly higher average values of glycaemia compared with severe group (7.67 ± 3.7 vs. 5.62 ± 0.9, p = 0.018). In the group with severe COPD, it was not confirmed any factor with significant predictive effect on the values of glycaemia. As independent significant factors that affect blood glucose in a group of very severe COPD were confirmed cholesterol (p <0.0001) and HDL (p = 0.018).

**CONCLUSION:**

These results suggest that the presence of the COPD in patients itself is a factor that results in the clinical presentation of diabetes mellitus Type 2.

## Introduction

Chronic obstructive pulmonary disease (COPD) is defined as abnormal inflammatory response of the lung to noxious gases and particles. The main epidemiological factor is smoking [1]. COPD is affecting nearly 300 million people worldwide and killing 3 million individuals each year [2]. Clinically, COPD is characterized by shortness of breath, and chronic, mostly productive cough that persists for weeks to months. All patients demonstrate airflow limitation on lung function tests with reduced expiratory flow rates. The main COPD feature is lung inflammation, which intensifies with disease progression [3].

Comorbidities in COPD are frequent; they influence the prognosis of patient’s diseases. The most common are cardiovascular diseases, skeletomuscular disorders, pulmonary malignant tumors, metabolic syndrome and many others, and have significantly impact on patients’ quality of life, exacerbation frequency, and survival, and they largely affect the prognosis of the disease [4, 5]. One of the COPD comorbidities is diabetes mellitus, as one of metabolic syndrome. Metabolic syndrome is defined as a complex of interrelated cardiovascular risk factors. It has clinically relevant negative effects on subjects exercise capacity, as well as on health status and mortality [7, 8].

Some authors considered COPD as a novel risk factor for new onset type 2 diabetes mellitus via multiple pathophysiological alterations first of all nonspecific low grade inflammation that occur in the lung and then split over in the whole body, and is responsible for systemic characteristics of the disease [8]. Systemic inflammation, with elevated markers such as C-reactive protein (CRP), tumor necrosis factor-α (TNF-α) and interleukin-6 (IL-6), plays an important role in both, the progression of COPD and the development of insulin resistance. Smoking is one cause of inflammation. Smokers have a two-fold higher risk of developing diabetes mellitus type 2 than nonsmokers [9].

The other reasons are: oxidative stress which is also considered as a reason for developing lung inflammation, insulin resistance [26] weight gain and alterations in metabolism of adipocytes. Type 2 DM is particularly common medical disorder and a leading cause of morbidity and mortality worldwide [10]. Disturbances in glucose metabolism are more frequent in COPD patients than in non COPD individuals. Similarly, almost half of all COPD patients suffer from other medical problems frequently linked to diabetes, such as elevated blood pressure and higher levels of cholesterol [11].

They are reasons for various cardiovascular complications in COPD patients. In many studies have found that DM causes an accelerated decline in lung functions as compared to non-diabetics. Diabetes especially uncontrolled is linked to worsening the outcomes such as (longer hospital stay and risk of death) in people that suffer from an exacerbation of COPD. The need to use of corticosteroid therapy during exacerbations can complicate the status of diabetes mellitus in those patients [12, 13].

The aim of the study was to investigate the prevalence of diabetes mellitus (Type 2) privies diagnosed COPD patients with severe and very severe stage of the disease, which was stable.

## Material and Methods

We investigated 100 subjects, all of them smokers, with smoking status >10 years. The duration of COPD in these patients was more than 9 years and diabetes mellitus more than 5 years. Subjects with COPD were stratified in two groups according to Global Initiative for Chronic Obstructive Lung Disease (GOLD). 64 of them were with severe stage of the disease: 50% > FEV1 ≥ 30%, FEV1/FVC < 0, 70, and 36 subjects with very severe stage of the disease: FEV1 < 30%, FEV1/FVC < 0, 70. It was clinical, randomized, cross sectional study. Besides demographic parameters (age, gender) and functional parameters, CRP (C-reactive protein) body mass index (BMI), cholesterol, LDL (low density lipoprotein) and HDL (high density lipoprotein), the level of blood sugar and HbA1c (Glycated hemoglobin (hemoglobin A1c) ware measured.

### Statistical analysis

Statistical analysis of the data base was made in the program SPSS for Windows 17, 0. Testing of the distribution of the data was done with Kolmogorov - Smirnov and Shapiro-Wilk’s test). Categorical variables were presented with absolute and relative numbers; numeric variables were shown MPC descriptive statistics (mean, median, rank values).

To test the significance of differences between the two COPD groups, were used parametric and nonparametric methods for independent samples (Chi-square test, Student-s test, Mann-Whitney U test). Categorical variables were presented with absolute and relative numbers; numeric variables were shown MPC descriptive statistics (mean, median, rank values). To test the significance of differences between the two COPD groups, were used parametric and nonparametric methods for independent samples (Chi-square test, Student-s test, Mann-Whitney U test). To determine the correlation between blood sugar and certain parameters was used Pearson-s coefficient of linear correlation). Multiple regression analysis was used to determine significant independent factors associated with blood sugar levels. For statistically significant values were taken at p < 0.05.

## Results

The prevalence of diabetes mellitus in our survey in total number of COPD patients with severe and very severe stage was 21%.

### Severe vs. very severe COPD

In the men significantly more likely than women were registered very severe COPD (45.45% vs. 17.65%, p = 0.006) (Table 1).

**Table 1:** The prevalence of gender in both groups of COPD patients

Gender	COPD (severe) N = 64	COPD (very severe) N = 36	p-value
Female n = 34	28 (82.35%)	6 (17.65%)	p = 0.006
Male n = 66	36 (54.55%)	30 (45.45%)

p (Chi-square test) p < 0.01.

Patients with severe and very severe COPD were insignificant different age and BMI (p = 0.75 and p = 0.14 consequently) (Table 2).

**Table 2:** The age, body mass index and functional parameters in both groups of patients

Variable	Group	Mean ± SD	Min - Max	p-value
Age (years)	Severe	62.53 ± 11.3	37 - 88	
	Very severe	61.83 ± 9.1	45 - 80	*p = 0.75
BMI (kg/m2)	Severe	24.22 ± 5.2	16 - 36	
	Very severe	25.9 ± 5.9	19.3 - 35	*p = 0.14
FVC L	Severe	1.78 ± 0.5	0.9 - 3.28	*p < 0.01
	Very severe	1.23 ± 0.4	0.56-1.89	
FVC%	Severe	56.25 ± 11.2	40 - 88	*p < 0.01
	Very severe	37.67 ± 12.9	20 - 67	
FEV1 L	Severe	1.02 ± 0.2	0.64-1.58	*p<0.01
	Very severe	0.67 ± 0.2	0.25-0.53	
FEV1%	Severe	40.6 ± 6.8	31-50	*p<0.01
	Very severe	23.3 ± 5.4	11-29	
FEV1/FVC%	Severe	60.84 ± 6.8	48 -70	*p < 0.01
	Very severe	52.61 ± 8.6	36 - 66	

* p (Student-s t-test).

### C-reactive protein (CRP)

In the group with very severe COPD were registered significantly higher values of C-reactive protein versus patients with severe COPD (p < 0.0001) (Table 3).

**Table 3:** The level of C-reactive protein in patients with severe vs very severe COPD

Variable	Group	Mean ± SD	Median (IQR)	p-value
	Severe	5.62 ± 1.6	5 (4.5 - 6.5)	
CRP mg/L	Very severe	9.28 ± 3.2	9 (7 - 10	*p < 0.0001

P (Mann- Whitney U test).

### Glycemia

In the group of patients with very severe COPD were recorded significantly higher average values of glycemia and HbA1c, compared with the group with severe COPD (7.67 ± 3.7 vs. 5.62 ± 0.9, p = 0.018). (Table 4, Table 5).

**Table 4:** The level of glycemia in patients with severe versus very severe COPD

Variable	Group	Mean ± SD	Min - Max	p-value
Glycemia (mmol/l)	Severe	5.62 ± 0.9	4.6 – 8.6	*p =0.018
	Very severe	7.67 ± 3.7	4.5 – 15.3	

* p (Student-s t-test).

**Table 5:** The level of HbA1c in patients with severe versus very severe COPD

Variable	Group	Mean ± SD	Median (IQR)	p-value
HbA1c %	Severe	5.6 ± 0.9	5.5 (.5 - 6.25)	*p < 0.0001
	Very severe	6.25 ± 1.8	5.25 (5–8.25)	

P (Mann- Whitney U test).

Men with severe and very severe COPD had insignificant higher average value of blood glucose than women (5.68 ± 0.9 vs. 5.54 ± 1.0 and 8.0 ± 3.6 vs. 6.03 ± 1.3 consequently) (Table 6).

**Table 6:** The average value of blood glucose in female vs. male in both groups

COPD Group	Gender	Descriptive statistic glycaemia		p-value

		Max ± SD	Min - Max
Severe	Female n=28	5.54 ±1.0	4.6 – 7.9	*p = 0.5
	Male n=36	5.68 ± 0.9	4.7 – 8.6	
Very severe	Female n=6	6.03 ± 1.3	4.7 – 7.7	*p = 0.2
	Male n=30	8.0 ± 3.6	4.5 – 15.3	

* p (Student-s t-test);

** p (Mann-Whitney U test).

In the group with severe COPD, HbA1c positively correlated with BMI (r = 0.318, p = 0.01), cholesterol (r = 0.239, p = 0.057) and LDL (r = 0.314 = 0.012), and significantly negatively correlates with HDL (r = - 0.433, p <0.001).

In the group with very severe COPD, cholesterol significantly positively correlated with LDL (r = 0.896, p <0.001) and HbA1c (r = 0.79, p <0.001), while significantly negatively correlated with the age of patients (r = - 0.458, p = 0.005) and the values of FVC% (r = - 0.37, p = 0.025) and HDL (r = - 0.793, p <0.001) (Table 7).

**Table 7:** The correlation of HbA1c with age, functional parameters, BMI, cholesterol, LDL, HDL

Correlation cholesterol /	r - Pearson	

	Severe	Very severe
Age (years)	r = 0.07, p=0.57	r = - 0.428, p=0.006
FVC L	r = 0.173, p=0.17	r = - 0.033, p=0.8
FVC%	r = 0.159, p=0.21	r = - 0.146, p=0.4
FEV1 L	r = - 0.05, p=0.69	r = - 0.065, p=0.7
FEV1 %	r = - 0.05, p=0.9	r = - 0.238, p=0.16
FEV1/FVC%	r = - 0.075, p=0.55	r = 0.07, p=0.68
BMI	r = 0.318, p=0.01	r = 0.144, p=0.4
Cholesterol (mmol/l)	r = 0.239, p=0.057	r = 0.792, p<0.001
LDL (mmol/l)	r = 0.314, p=0.012	r = 0.688, p<0.001
HDL (mmol/l)	r = - 0.433, p<0.001	r = - 0.793, p<0.001

Results from Multiple regression analysis which investigate the impact of BMI, LDL and HDL glucose, in the group with severe COPD, (which bivarijant analysis proved to be significantly related to blood glucose) not confirmed any factor with significant predictive effect on the values of glycemia.

**Figure 1:**
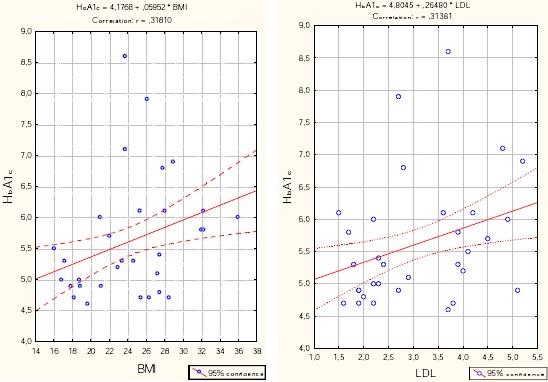
*Correlation – HbA1c vs. BMI and LDL in the group with severe COPD*.

As independent significant factors that affect blood glucose in a group of very severe COPD confirmed cholesterol (p <0.0001) and HDL (p = 0.018).

With increasing values of cholesterol to 1mmol/l, the values of average glycemia increased to 2.798 (B = 2.798), while an increase in HDL values at 1 mmol/l, the values of average blood glucose increase to 3.489 (B = 3.489).

**Figure 2:**
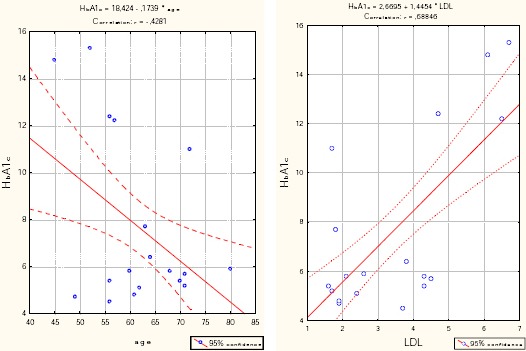
*Correlation - HbA1c vs. LDL and age in the group with very severe COPD*.

These results suggest that the presence of the COPD in patients itself is a factor that results in the clinical presentation of diabetes mellitus Type 2. The patients with very severe COPD more likely have diabetes mellitus Type 2. Results from Multiple regression analysis which investigate the impact of BMI, LDL and HDL glucose, in the group with severe COPD, (which bivariate analysis proved to be significantly related to blood glucose) not confirmed any factor with significant predictive effect on the values of glycaemia. As independent significant factors that affect blood glucose in a group of very severe COPD confirmed cholesterol (p <0.0001) and HDL (p = 0.018).

**Figure 3:**
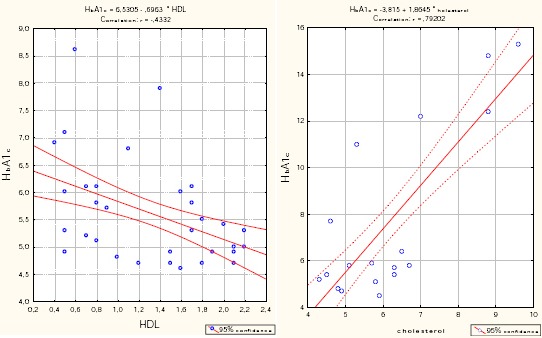
*Correlation - HbA1c vs. HDL and cholesterol in the group with a very severe COPD*.

## Discussion

Disturbances in glucose metabolism are more common in COPD patients than in subjects without COPD. COPD, metabolic syndrome and diabetes mellitus type 2 are associated with advanced age. It is well known that offspring of affected parents are more likely to develop both COPD and type 2 diabetes mellitus. Smoking during pregnancy can in part explain the association between COPD and type 2 diabetes mellitus [11, 12].

According to data from the International Federation and diabetes data of the National Registry of diabetes, incidence of the disease in Macedonia in 2012 was 6.9 %, and the total number of diagnosed persons with diabetes was 119 200 [14].

**Table 8:** Multiple regression analysis of the impact of BMI, LDL and HDL on the glucose in the group with severe COPD

Coefficients							

Model	Unstandardized Coefficients		Standardized Coefficients	t		95% Confidence Interval for B	
	
	B	Std. Error	Beta		Sig.	Lower Bound	Upper Bound
1 (Constant)	5.636	1.304		4.323	0.000	3.028	8.243
BMI	0.029	0.026	0.155	1.137	0.260	-0.022	0.080
LDL	-0.570	0.344	-0.355	-1.655	0.103	-1.259	0.119
HDL	0.009	0.164	0.010	0.053	0.958	-0.318	0.336

a) Dependent Variable: glycemia.

In our survey the prevalence of Diabetes mellitus type 2 in COPD patients was 21%.

According study from 2015, Paola Rogliani, the prevalence between diabetes and COPD varies between studies reported; however it is known that diabetes affects 2–37 % of patients with COPD [14].

**Table 9:** Multiple regression analysis of the impact of age, LDL, HDL cholesterol and glucose in a very severe group COPD

Coefficients							

Model	Unstandardized Coefficients		Standardized Coefficients			95% Confidence Interval for B	
	
	B	Std. Error	Beta	t	Sig.	Lower Bound	Upper Bound
1 (Constant)	-18.160	7.696		-2.360	0.025	-33.856	-2.464
Age	0.062	0.060	0.154	1.049	0.302	-0.059	0.184
Cholesterol	2.798	0.623	1.189	4.489	0.000	1.527	4.069
LDL	0.276	0.511	0.131	0.540	0.593	-0.767	1.318
HDL	3.489	1.399	0.560	2.493	0.018	0.635	6.343

^a^) Dependent Variable: glycemia.

The reported prevalence of diabetes among patients with COPD ranges from 1.6 to 16%. As in COPD, smoking has been established as a risk factor for diabetes, quitting for more than 5 to 10 years mitigates that risk. Type 2 diabetes is more prevalent in moderate-to-very severe (but not mild) COPD than in the general population, with an overall prevalence of 12.7% in the combined ARIC and CHS cohorts and 12.2% in The Health Improvement Network dataset. The evidence for an interaction between diabetes and COPD is supported by studies that demonstrate reduced lung function as a risk factor for the development of diabetes. Inflammatory mediators such as TNF-α, IL-6, and CRP, which are elevated in COPD, are also increased in diabetes [10]. The impact of parental use of corticosteroids on the management of diabetes during COPD exacerbations and the effect of diabetes control on COPD outcomes is of great clinical concern. Mortality was found to be significantly higher in patients having poor glycemic control who were hospitalized for acute COPD exacerbation, and even after discharge, diabetes remained a risk factor for mortality. It is uncertain if tighter and better glucose control can improve COPD outcome [7].

The level of C-reactive protein in our patients with very severe stage of COPD was significantly higher versus patients with severe COPD (p < 0.0001).

According Gregory L Kinney, people with diabetes mellitus have a 22% increased risk of developing chronic obstructive pulmonary disease, whereas those with chronic obstructive pulmonary disease have a 40–100% increased risk of developing diabetes [16].

In the United States they have estimated a prevalence of diabetes of 12.7% to 16.3% among patients with COPD, significantly higher than in the general population. Longitudinal studies have confirmed that COPD is a risk factor for incident diabetes [17].

A recent review of the literature established the complex link between smoking and obesity in the development of co-morbidities, involving an enzyme cascade that originates in adipose tissue considered a site for production of cytokines (TNF-a, IL-6, etc), while adiponectin decreases with increased adiposity. This increases insulin resistance, circulating free radicals and oxidative stress, exacerbating the initial pulmonary inflammation. Adipose tissue stimulation is promoted by tissue hypoxia, smoking and the degree of bronchial obstruction. Severity of systemic inflammation is a direct measure of severity of COPD. In Vinay Mahishale study, more than two-third of the subjects had moderate to severe COPD who had DM, which is in agreement with many cohort studies which have demonstrated that moderate to severe COPD increases the risk of DM (OR 1.4 and 1.5, respectively) [18-21].

Conversely, in a US cohort, the relative risk of developing COPD was higher (HR 1.22) in patients with diabetes than in non-diabetics. It is well known fact that DM significantly affects the outcome of COPD including time to first hospitalization and 5-years mortality rates. According to the Emerging Risk Factors Collaboration, the HR for COPD-related death was 1.27 as compared to subjects without diabetes. Another study showed that an increase in blood glucose of 1 mmol/L increases the risk of death by 15%. Parrapil et al. and Baker et al., confirmed an increased risk of death (OR 1.93) and hospital stays were 10.3% longer for patients with diabetes hospitalized for COPD exacerbation [22-25].

Prevalence of DM in COPD patients in Mannino DM, study is 25.63% when actively screened in tertiary care hospital. It is feasible and imperative to screen all COPD patients for DM in all health care facilities routinely [23, 26].

In study of Zareen Kiran, forty COPD patients were compared with thirty five age match controls. HOMA-IR (insulin resistance) was found to be higher in cases as compared to controls (2.85 v/s 2.00) with a p value <0.000 [27]

In conclusion, the definition of COPD as primarily a lung disease has been changed, and now the broader definition of COPD as a systemic inflammatory syndrome has been proposed. There is increasing evidence that another disease occur in greater frequency amongst patients with COPD than in the general population, and that these comorbidities significantly impact on patient outcomes. Evidence for this approach has been provided by strong associations with increased rates especially with cardiovascular diseases, metabolic syndrome, anemia, musculoskeletal disease and malignances. From the results of our survey COPD itself is a factor that leads to clinical presentation of diabetes mellitus Type 2.

## References

[1] Global Initiative for Chronic Obstructive Lung Disease (GOLD) Global strategy for the diagnosis, management and prevention of chronic obstructive pulmonary disease.

[2] Mathers D, Loncar D (2006). Projections of global mortality and burden of disease from 2002 to 2030. PLoS medicine.

[3] Barnes P (2000). Chronic Obstructive Pulmonary Disease. N Engl J Med.

[4] Manzotti E, Barclay L, Patel A, Hurst J (2011). Extrapulmonary comorbidities in chronic obstructive pulmonary disease: state of the art. Expert Rev Respir Med.

[5] Barnes P (2000). Chronic Obstructive Pulmonary Disease. N Engl J Med.

[6] Barnes PJ (2010). Chronic obstructive pulmonary disease: effects beyond the lungs. PLoS Med.

[7] Chatila W, Thomashow B, Minai O, Criner G, Make B (2008). Comorbidities in Chronic Obstructive Pulmonary Disease. Proc Am Thorac Soc.

[8] Naik D, Joshi A, Vizhalil Paul V, Thomas N (2014). Chronic obstructive pulmonary disease and the metabolic syndrome: Consequences of a dual threat. Indian J Endocrinol Metab.

[9] Young J, Sin D (2012). Lung inflammation in COPD: why does it matter?. F1000 Med Rep.

[10] Maumus S, Marie B, Siest G (2005). PHARMD, Visvikis-Siest S. A Prospective Study on the Prevalence of Metabolic Syndrome Among Healthy French Families Two cardiovascular risk factors (HDL cholesterol and tumor necrosis factor-α) are revealed in the offspring of parents with metabolic syndrome. Diabetes Care.

[11] Breyer K, Spruit A, Hanson K, Franssen M, Vanfleteren E, Groenen T, Bruijnzeel L, Wouters F, Rutten P (2014). Prevalence of metabolic syndrome in COPD patients and its consequences. PLoS One.

[12] Mahishale V, Mahishale A, Patil B, Sindhuri A, Eti A (2015). Screening for diabetes mellitus in patients with chronic obstructive pulmonary disease in tertiary care hospital in India. Niger Med J.

[13] Phillips C (2013). Nutrigenetics and Metabolic Disease: Current Status and Implications for Personalised Nutrition Nutrients. http://dx.doi.org/10.1201/b16307-3.

[14] International Diabetes Federation and data from the National Registry of diabetes Official Gazette of Republic of Macedonia 43/2012 and 145/2012.

[15] Rogliani P, Lucà G, Lauro D (2015). Chronic obstructive pulmonary disease and diabetes. COPD Research and Practice.

[16] Kinney G, Baker E (2014). Type 2 diabetes mellitus and chronic obstructive pulmonary disease: need for a double-pronged approach.

[17] Barnes P, Celli B (2009). Systemic manifestations and comorbidities of COPD. Eur Respir J.

[18] Mirrakhimov M (2012). Chronic obstructive pulmonary disease and glucose metabolism: a bitter sweet symphony. Cardiovasc Diabetol.

[19] Kaur J (2014). A Comprehensive Review on Metabolic Syndrome. Cardiology Research and Practice, Review Article. 2014.

[20] Rimm B, Manson E, Stampfer J, Colditz A, Willett C, Rosner B (1993). Cigarette smoking and the risk of diabetes in women. Am J Public Health.

[21] Cavaillès A, Brinchault-Rabin G, Dixmier A, Goupil F, Gut-Gobert C, Marchand-Adam S (2013). Comorbidities of COPD. Eur Respir Rev.

[22] Mahishale V, Mahishale A, Patil B, Sindhuri A, Et A (2015). Screening for diabetes mellitus in patients with chronic obstructive pulmonary disease in tertiary care hospital in India. Niger Med J.

[23] Hansell L, Walk A (2003). What do chronic obstructive pulmonary disease patients die from? A multiple cause coding analysis. Eur Respir J.

[24] Directorate General of Health Services, India National programme for prevention and control of cancer, diabetes, cardiovascular disease and stroke (NPCDCS).

[25] Rana S, Mittleman A, Sheikh J (2004). Chronic obstructive pulmonary disease, asthma, and risk of type 2 diabetes in women. Diabetes Care.

[26] Mannino M, Thorn D, Swensen A, Hulguin F (2008). Prevalence and outcomes of diabetes, hypertension and cardiovascular disease in COPD. Eur Respir J.

[27] Kiran K, Majeed N, Zuberi B (2015). Comparison of frequency of insulin resistance in patients with chronic obstructive pulmonary disease with normal controls. Pak J Med Sci.

